# ﻿Emending *Gymnopus* sect. *Gymnopus* (Agaricales, Omphalotaceae) by including two new species from southern China

**DOI:** 10.3897/mycokeys.87.76125

**Published:** 2022-03-04

**Authors:** Ji-Peng Li, Vladimír Antonín, Genevieve Gates, Lu Jiang, Tai-Hui Li, Yu Li, Bin Song, Chun-Ying Deng

**Affiliations:** 1 State Key Laboratory of Applied Microbiology Southern China, Guangdong Provincial Key Laboratory of Microbial Culture Collection and Application, Institute of Microbiology, Guangdong Academy of Sciences, Guangzhou, Guangdong 510070, China Jilin Agricultural University Changchun China; 2 Engineering Research Center of Chinese Ministry of Education for Edible and Medicinal Fungi, Jilin Agricultural University, Changchun, Jilin 130118, China Institute of Microbiology, Guangdong Academy of Sciences Guangzhou China; 3 Department of Botany, Moravian Museum, Zelný trh 6, Brno CZ-659 37, Czech Republic Moravian Museum Brno Czech Republic; 4 Tasmanian Institute of Agriculture, Private Bag 98, Hobart, Tasmania 7001, Australia Tasmanian Institute of Agriculture Hobart Australia; 5 Shenzhen Wildlife Conservation Division, Shenzhen, Guangdong 518048, China Shenzhen Wildlife Conservation Division Shenzhen China; 6 Guizhou institute of biology, Guizhou Academy of Sciences, Guiyang, Guizhou 550009, China Guizhou institute of biology, Guizhou Academy of Sciences Guiyang China

**Keywords:** Morphology, new taxa, phylogeny, taxonomy

## Abstract

Based on phylogenetic analyses, some newly studied Chinese mushroom specimens were found to represent two distinct species within the genus *Gymnopus*. Along with *G.fusipes* (sect. Gymnopus) they form a distinct clade with high support, although their macromorphological characters seem to be closer to members of *Gymnopus* sect. Levipedes or sect. Vestipedes (Collybiopsis). When examined in detail, their micromorphological characters, especially the type of pileipellis, support them as new members of *G.* sect. Gymnopus. Therefore, two new species, *G.omphalinoides* and *G.schizophyllus*, and the emended circumscription of sect. Gymnopus are proposed in this paper. Detailed morphological descriptions, colour photos, illustrations of the two new species, morphological comparisons with similar taxa and the molecular-phylogenetic analyses of the combined nrITS and nrLSU data are presented. A key to the known species of *G.* sect. Gymnopus is also presented.

## ﻿Introduction

*Gymnopus* (Pers.) Roussel sect. Gymnopus is a monotypic section and its type species, *Gymnopusfusipes* (Bull.) Gray, also typifies the genus (Antonín and Noordeloos 2010). The sectional name, therefore, was proposed automatically. Formerly, *G.fusipes* was placed in *Collybia* (Fr.) Staude sect. Striipedes (Fr.) Quél. as *C.fusipes* (Bull.) Quél. (Singer 1986). Based on morphology, several species, in fact, several sections, were moved from *Collybia* to *Gymnopus*, a genus that was defined mainly based on American and European material (Antonín et al. 1997). Since then, the character of the pileipellis, especially the terminal cells, has become a significant factor in the delimitation of the sections within the genus. After undergoing a series of revisions, *Gymnopus* sensu lato (s.l.) was restricted as a monophyletic genus (*Gymnopus* sensu stricto (s. str.)) that comprised four sections. The other three sections are G.sect.Androsacei (Kühner) Antonín & Noordel, sect. Impudicae (Antonín & Noordel.) Antonín & Noordel. and sect. Levipedes (Quél.) Halling (Oliveira et al. 2019).

Morphologically, the current circumscription of G.sect.Gymnopus was adopted from Clémençon (1981) as Collybiasect.Striipedes. As a monotypic section, its circumscription is dominated by its type species which is characterised by a fleshy pileus, fusoid stipe with a distinct pseudorrhiza and a pileipellis made up of inflated, irregular, often coralloid elements, similar to the *Dryophila*-type structure (Antonín and Noordeloos 2010; Oliveira et al. 2019). It stands in stark contrast to other sections. Many studies published in recent years with an emphasis on *Gymnopus* reported or described species from the other sections, and discussions relating to the type species or G.sect.Gymnopus were hardly addressed. Wilson and Desjardin (2005) and Mata et al. (2007) noted that *G.fusipes* and members of G.sect.Levipedes share a similar pileipellis and that the type species of the genus mainly differs in the stipe with a pseudorrhiza. Besides, only *Collybiasubsulcatipes* A.H. Sm. was considered a probable member of G.sect.Gymnopus based on morphology (Antonín and Noordeloos 1997, as *Collybiasulcatipes* A.H. Smith). It is characterised by a smooth or longitudinally grooved to subsulcate stipe with a long pseudorrhiza (Smith 1944). Nevertheless, whether this species belongs to this section is difficult to confirm because of the lack of molecular data.

Phylogenetically, Mata et al. (2004) reported on the phylogenetic position of *G.fusipes* and showed that it forms a distinct clade that is closely related to *Setulipesandrosaceus* (L.) Antonín and always among other clades dominated by *Gymnopus* taxa. Wilson and Desjardin (2005) also produced a similar phylogenetic result. As the species typified the genus, these results had repercussions on the generic relationships. Hence, *S.androsaceus* was transferred to *Gymnopus* (Mata et al. 2004) and was designated as the type species of G.sect.Androsacei (Noordeloos and Antonín 2008). Subsequently, Oliveira et al. (2019) used a multi-gene phylogenetic analysis to restrict the concept of genus *Gymnopus* and to further confirm that G.sect.Androsacei is the closest group to G.sect.Gymnopus. However, there was no update on the phylogenetic nature of G.sect.Gymnopus due to the lack of new material.

In this study, two new species of G.sect.Gymnopus are described based on morphology and phylogenetic analysis. Detailed morphological descriptions, colour photos, illustrations of the species, morphological comparisons with similar taxa and molecular-phylogenetic analyses of combined nuclear ribosomal internal transcribed spacer (nrITS) and nuclear ribosomal large subunit (nrLSU) data are presented. An emended circumscription and a key to the species of G.sect.Gymnopus are provided.

## ﻿Material and methods

### ﻿Abbreviations

For Latin names: ***G.*** = *Gymnopus*; ***Ma.*** = *Marasmius*; ***Mi.*** = *Micromphale*; ***My.*** = *Mycetinis*; ***P.*** = *Paragymnopus*.

For phylogenetic analysis: **ML** = Maximum Likelihood; **BI** = Bayesian Inference; **BP** = Bootstrap Proportions; **PP** = Posterior Probability.

For collection locality: **FNNR** = Fanjingshan National Nature Reserve; **MC** = Maguan County; **MR** = Meizihu Reservoir; **TFP** = Tianluhu Forest Park; **WSA** = Wutongshan Scenic Area; **YNNR** = Yunkaishan National Nature Reserve.

For climate: **AAT** = average annual temperature; **AAR** = average annual rainfall; **MST** = major soil type; **MMMM** = mid-subtropical mountain moist monsoon; **SEM** = subtropical eastern monsoon; **SM** = subtropical monsoon; **SSM** = south subtropical monsoon; **SSO** = south subtropical oceanic.

﻿For soil type: B = brown; DBS = dark brown soil; La = laterite; LRS = lateritic red soil; MSMS = mountain shrub meadow soils; MRS = mountain red soil; RS = red soil; YBS = yellow brown soil; YS = yellow soil.

### ﻿Specimen collection and drying treatment

Nine collections from China were examined in this study: one came from the Guizhou Province (Tongren City), three collections from the Yunnan Province (one from Pu’er City and two from Maguan County) and five collections from the Guangdong Province (one from Guangzhou City, one from Shenzhen City and three from Xinyi City). The exact localities and their environmental characteristics are shown in Table 1. The fresh basidiomata of each collection were wrapped in separate mesh bags and dried in an electric drier operated below 50 °C. Dried collections were deposited in the Fungarium of Guangdong Institute of Microbiology, China (**GDGM**), Fungarium of the Herbarium of Kunming Institute of Botany, Chinese Academy of Sciences (**KUN-HKAS**) or Herbarium Mycology of Jilin Agricultural Science and Technology University (**HMJU**). The herbarium abbreviations follow Thiers (2021).

**Table 1.** The environmental characteristics of localities for each collection.

Locality	Climate	Average annual temperature	Average annual rainfall	Major soil type	References
FNNR	MMMM	16.9 °C	1351 mm	YS	Xiao et al. 1998; Zhong et al. 2011
MC	SEM	16.9 °C	1345 mm	La, LRS, RS, YS, YBS, BS, DBS	Zhao 2007
MR	SM	17.8 °C	1514.6 mm	La, LRS, RS	Tao 2002, 2006
TFP	SSM	22 °C	1725 mm	LRS	Huang and Li 2006; Kong et al. 2013
WSA	SSO	22.4 °C	1948.4 mm	LRS, RS, MSMS	Xv et al. 2009; Zhou et al. 2011
YNNR	SSM	18 °C	2300–2600 mm	LRS, MRS, YS	Huang (1998); Li et al. 2021b

### ﻿Morphological studies

Fresh basidiomata were photographed and used for macromorphological descriptions. The colours are coded from Kornerup and Wanscher (1978). The ecology of the specimens is presented below. Lamellae were counted where ‘L’ refers to the number of full-length lamellae and ‘l’ refers to the number of lamellulae tiers.

Micromorphological structures were observed via a ZEISS Axio Lab. A1 microscope based on the hand-made sections of dried basidiomata mounted in 5 % KOH on a glass slide. When necessary, Congo Red solution was used as a stain and Melzer’s reagent was used to test amyloid or dextrinoid reactions. For the various microscopic structures, ‘n’ refers to the number of measured elements. For basidiospores, ‘E’ represents the quotient of length and width in any one spore, and ‘Q’ represents the mean of E values. Basidiospore measurements do not include apiculus and are presented as ‘(a)b–c(d)’, where ‘b–c’ represents the minimum of 90 % of the measured values and ‘a’ and ‘d’ represent the extreme values. The main body (sterigmata or excrescences not included) of basidia, basidioles, pleurocystidia and cheilocystidia were measured (if present).

### ﻿DNA extraction, amplification and sequencing

Genomic DNA was extracted from dried tissue via a Magen HiPure Fungal DNA Kit (Magen Biotech Co., Ltd., Guangzhou) Fungal DNA Kit as in Li et al. (2021a). The nrITS (the nuclear ribosomal internal transcribed spacer) region and the nrLSU (nuclear ribosomal large subunit) gene were amplified by the polymerase chain reaction (PCR) technique using the primers ITS5 and ITS4 (nrITS; White et al. 1990), and LR0R and LR5 (nrLSU; Vilgalys and Hester 1990; Cubeta et al. 1991), respectively. A common PCR programme was used for amplification of both markers and is given below: 4 min at 95 °C; 35 cycles of 45 s at 95 °C, 45 s at 53 °C, 60 s at 72 °C; 10 min at 72 °C. Amplified products were used for Sanger dideoxy sequencing performed by Beijing Genomics Institute (BGI). The newly generated sequences were assembled from two overlapping reads and trimmed via BioEdit v.7.0.9 (Hall 2011). Before depositing in GenBank (Sayers et al. 2021; Table 2), quality control was done following the methods in Nilsson et al. (2012).

**Table 2.** Information on DNA sequences used in the phylogenetic analyses. Newly generated sequences are highlighted in bold and type specimen is marked with an asterisk (*).

Taxon name	ITS	LSU	Collection No.	Locality	Reference
*Agaricales* sp.	AB859204	AB859204	Sw2-1	Japan	GenBank
*G.adventitius* nom. prov.	KY026760	KY026760	SFSU:DED8813	Not given	Petersen and Hughes (2016)
*G.alliifoetidissimus*	MT023348	MT017526	GDGM 76695	China	Li et al. (2021a)
*G.androsaceus*	KY026750	KY026750	CULTENN5609	USA	Petersen and Hughes (2016)
*G.androsaceus*	MH857175	MH868714	CBS 240.53	France	Vu et al. 2019
*G.androsaceus*	MH857174	MH868713	CBS 239.53	France	Vu et al. 2019
*G.androsaceus*	KY026748	KY026748	CULTENN5021h2	Canada	Petersen and Hughes (2016)
*G.androsaceus*	KY026663	KY026663	TENN:F-59594	Russia	Petersen and Hughes (2016)
*G.atlanticus*	KT222654	KY302698	URM 87728	Brazil	Coimbra et al. (2015)
*G.aurantiipes*	AY263432	AY639410	SFSU:AWW118	Indonesia	Wilson et al. (2004)
*G.brunneiniger*	MT232388	MW187069	XAL: Cesar50	Mexico	César et al. (2020)
*G.brunneodiscus*	MH589973	MH589988	BRNM 714974	South Korea	Ryoo et al. (2020)
*G.cremeostipitatus*	KF251071	KF251091	BRNM 747547	South Korea	Antonín et al. (2014)
*G.densilamellatus*	KP336685	KP336694	BRNM 714927	South Korea	Ryoo et al. (2016)
*G.dryophiloides*	MH589967	MH589985	BRNM 781447	South Korea	Ryoo et al. (2020)
*G.dryophilus*	DQ241781	AY640619	TENN:F-57012	Not given	Matheny et al. (2006)
*G.dysodes*	KY026666	FJ750265	TENN:F-61125	USA	Hughes and Petersen (2016)
*G.foetidus*	KY026739	KY026739	TENN:F-69323	USA	Hughes and Petersen (2016)
*G.frigidomarginatus* nom. prov.	KY026648	KY026648	TENN:F-55679	USA	Hughes and Petersen (2016)
*G.fusipes*	AY256711	AY256711	TENN:F-59300	Austria	Mata et al. (2004)
*G.fusipes*	KY026727	KY026727	TENN:F-69254	Slovakia	Hughes and Petersen (2016)
*G.fusipes*	AY256710	AY256710	TENN:F-59217	France	Mata et al. (2004)
*G.impudicus*	LT594119	LT594119	BRNM 714849	Czech Republic	Ryoo et al. (2016)
*G.inflatotrama* nom. prov.	KY026619	KY026619	TENN:F-48143	USA	Hughes and Petersen (2016)
*G.inflatotrama* nom. prov.	KY026744	KY026744	TFB 4529	USA	Hughes and Petersen (2016)
*G.inflatotrama* nom. prov.	KY026640	KY026640	TENN:F-53490	USA	Hughes and Petersen (2016)
*G.inflatotrama* nom. prov.	KY026632	KY026632	TENN:F-51233	USA	Hughes and Petersen (2016)
*G.inusitatus*	JN247553	JN247557	BCN:SCM B-4058	Spain	Antonín et al. (2012)
*G.iocephalus*	DQ449984	KY019630	TENN:F-52970	USA	Mata et al. (2007)
*G.irresolutus*	MF100973	Unavailable	SFSU:DED 8209	São Tomé	Desjardin and Perry (2017)
*G.montagnei*	DQ449988	AF261327	JMCR 143	Not given	Mata et al. (2007)
*G.neobrevipes*	MH673477	MH673477	TENN:F-14505	USA	Petersen and Hughes (2019)
*G.novae-angliae* nom. prov.	KY026745	KY026745	CULTENN4975	USA	Hughes and Petersen (2016)
*G.novomundi* nom. prov.	KY026759	KY026759	SFSU-DED5097	USA	Hughes and Petersen (2016)
*G.ocior*	KY026678	KY026678	TENN:F-65135	Belgium	Hughes and Petersen (2016)
***G.omphalinoides* sp. nov. **	**MW134044**	**MW134730**	*GDGM 78318	China	This study
***G.omphalinoides* sp. nov. **	**MW134047**	**MW134733**	HMJU 00506	China	This study
***G.omphalinoides* sp. nov. **	**MW134040**	**MW134726**	GDGM 44411	China	This study
***G.omphalinoides* sp. nov. **	**MW134045**	**MW134731**	GDGM 78483	China	This study
***G.omphalinoides***sp. nov.	**OK087326**	Unavailable	KUN-HKAS 107312	China	This study
*G.pallipes*	MW582856	**OK087327**	GDGM 81513	China	Li et al. (2021b) and this study
*G.portoricensis*	KY026627	KY026627	TENN:F-50999	Puerto Rico	Hughes and Petersen (2016)
***G.schizophyllus* sp. nov. **	**MW134041**	**MW134727**	GDGM 76287	China	This study
***G.schizophyllus* sp. nov. **	**MW134042**	**MW134728**	GDGM 77038	China	This study
***G.schizophyllus* sp. nov. **	**MW134043**	**MW134729**	*GDGM 77165	China	This study
***G.schizophyllus* sp. nov. **	**MW134046**	**MW134732**	KUN-HKAS 96494	China	This study
*G.similis*	KP336690	KP336697	BRNM 714981	South Korea	Ryoo et al. (2016)
*G.spongiosus*	KY026686	KY026686	TENN:F-65912	USA	Hughes and Petersen (2016)
*G.subsupinus*	KM975399	KM975375	PDD:96595	New Zealand	GenBank
*G.talisiae*	KT222655	KX958401	URM 87730	Brazil	Coimbra et al. (2015)
*Ma.androsaceus*	JN943605	JN941145	Sara Landvik:NN008037	Sweden	Antonín et al. (2014)
*Ma.androsaceus*	AF519893	AF519891	MUCL35155	Not given	Klonowska et al. (2013)
*Ma.otagensis*	MT974597	MT974601	PDD:106823	New Zealand	GenBank
*Ma.otagensis*	MT974600	MT974602	PDD:113265	New Zealand	GenBank
*Mi.foetidum*	KP877447	Unavailable	NEHU.MBSRJ.48	India	Borthakur and Joshi (2016)
*My.alliaceus*	KY696752	KY696752	TENN:F-55630	Russia	Petersen and Hughes (2017)
*My.scorodonius*	KY696748	KY696748	TENN:F-53474	USA	Petersen and Hughes (2017)
*Pa.perforans*	KY026625	KY026625	TENN:F-50319	Sweden	Petersen and Hughes (2017)

### ﻿Phylogenetic analyses

Representative species and their sequences were selected to cover all sections of *Gymnopus* s. str. based on recent publications (Mata et al. 2004; Petersen and Hughes 2016; Oliveira et al. 2019; César et al. 2020). In addition, four sequences annotated as *Marasmiusotagensis* were added to the matrix following an unpublished phylogenetic tree provided by Dr Jerry Cooper (Landcare Research, New Zealand). Two species of *Mycetinis* Earle were selected as the outgroup according to the phylogenetic results of Oliveira et al. (2019), Li et al. (2021a) and Li et al. (2021b). Our two-marker dataset, composed of ITS1-5.8S-ITS2-LSU sequences, was partitioned and used for the phylogenetic analyses. The samples NEHU MBSRJ48, HAKS 107312 and SFSU:DED 8209 have only ITS sequences available, and their LSU data were treated as missing data in the dataset. Information on sequences used in the phylogenetic analysis of this study is shown in Table 2. Sequences of each marker (nrITS and nrLSU) were aligned using MAFFT v.7.313 (Katoh and Standley 2013), applying the L-INS-I strategy, and manually concatenated and adjusted in BioEdit v.7.0.9 (Hall 2011). The combined dataset comprised four partitions (ITS1, the 5.8S gene, ITS2 and the LSU gene) and was analysed in the Maximum Likelihood (ML) and Bayesian Inference (BI) methods. The ML analysis was performed in RAxML v.8.2.10 (Stamatakis 2014), and the BI analysis was performed in MrBayes v.3.2.6 (Ronquist et al. 2012). The optimal substitution model for BI analysis was chosen by Modelfinder (Kalyaanamoorthy et al. 2017) using the Bayesian Information Criterion (BIC). The ML analysis was conducted using the GTRGAMMA substitution model, applying rapid bootstrap algorithm, with 5000 replicates. The BI analysis was implemented using two runs with four chains each for ten million generations sampling every hundredth generation. The average standard deviation of split frequencies was examined to make sure that the value was below 0.01. After discarding the first 25 % of trees as burn-in, a 50% majority rule consensus tree was generated from the remaining trees. Convergence of the MCMC chains was visualised in Tracer v. 1.7.1 (Rambaut et al. 2018) and examined manually. The tree files were viewed and edited in FigTree v1.4.3 (Rambaut 2009). The multiple sequence alignment and the ML and BI tree files were deposited in TreeBASE as Study ID 28774 (https://www.treebase.org).

## ﻿Results

### ﻿Phylogenetic results

A BLAST search of nrITS sequences revealed that a sequence annotated as “*Micromphalefoetidum*” (KP877447) was the most similar (7–8 different sites or more than 98.16% similarity) to the two new species described in this study.

**Figure 1.**
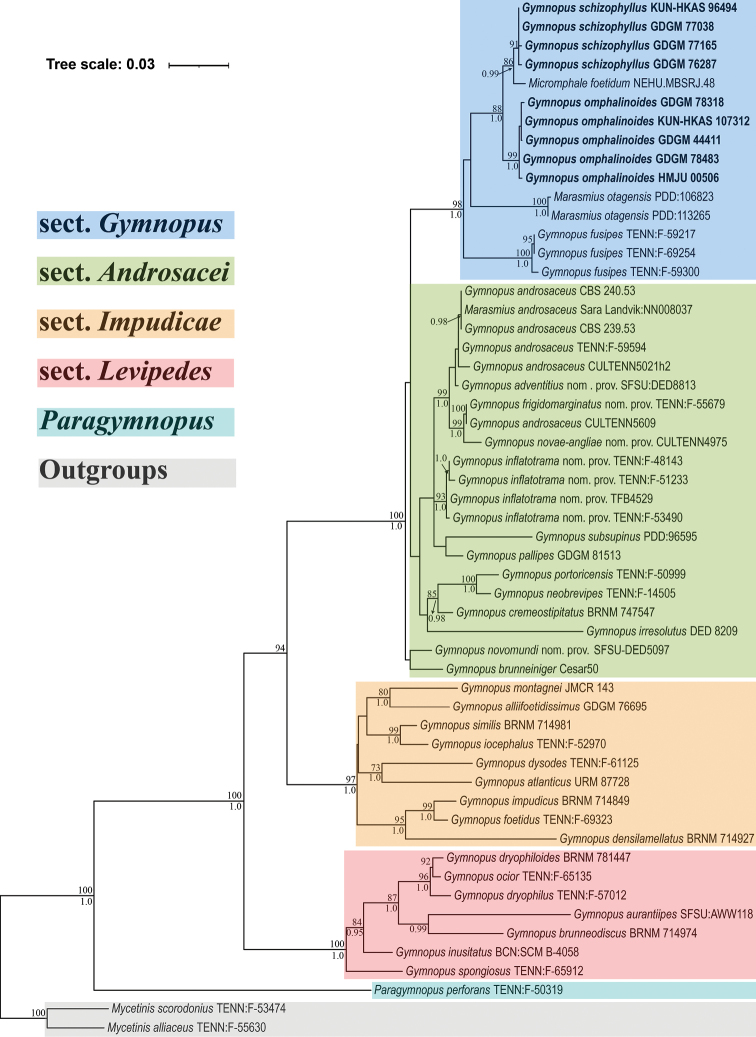
Phylogram generated by ML analysis of the combined dataset (ITS1-5.8S-ITS2-LSU region). ML-BP ≥ 70 % and BI-PP ≥ 0.95 are shown above and below the branches, respectively.

The combined dataset comprised 113 sequences including 58 nrITS and 55 nrLSU. The alignment is 1,716 bases long, of which 1,263 are constant sites, 139 are variable and parsimony-uninformative sites and 314 (18 %) are parsimony-informative sites. The best-fit model for each partition applied in the BI analysis was HKY+F+I+G4 (for the nrITS1, nrITS2 and nrLSU markers) and K2P (for the nr5.8S gene). ML and BI analyses produced nearly identical topologies and only the ML phylogram is presented (Fig. 1). The ML-BP and BI-PP support values are shown above and below the branches, respectively.

In the generated phylogenetic tree (Fig. 1), *Gymnopus* s. str. formed a strongly supported clade (BI-PP/ML-BP = 1.00/100 %). Inside this clade, four samples from China (GDGM 76287, 77038, 77165 and KUN-HKAS 96494) of one morphospecies and five samples from China (GDGM 44411, 78318, 78483, KUN-HKAS 107312 and HMJU 00506) of the other morphospecies grouped in two different lineages implying two distinct species within *Gymnopus* s. str. The nine samples from China along with a sample from India (NEHU MBSRJ48) formed a single clade with high support (BI-PP/ML-BP = 1.0/88 %). This clade and two samples from New Zealand (PDD: 106823, 113265) grouped in one clade as sister to *G.fusipes* (G.sect.Gymnopus). Furthermore, they formed a distinct group as a monophyletic clade with high support (BI-PP/ML-BP = 1.00/98 %).

## ﻿Taxonomy

### ﻿ Gymnopus omphalinoides

Taxon classificationFungiAgaricalesOmphalotaceae

J.P. Li, T.H. Li & Y. Li, sp. nov.

FE4ED886-1712-501B-9D97-9EABE5BE609C

837641

Figs 2 , 3

#### Typification.

China, Guangdong Province, Shenzhen City, Wutongshan Scenic Area, 16 September 2019, H. Huang, L.Q. Wu & N. Zhan (GDGM 78318, holotype!).

#### Etymology.

The epithet ‘*omphalinoides*’ (Lat.) refers to the omphalinoid or *Omphalina*-like basidiomata of the new species.

#### Diagnosis.

Differs from *G.volkertii* Murrill in its striate or grooved pileus and smaller basidiospores (4.0–5.5 × 2.5–3 μm). Basidiomata mainly gregarious on decayed wood in broadleaf forest; pileus disc reddish orange to dark brown becoming paler with age; lamellae broad, adnate and ventricose; stipe glabrous.

#### Description.

Basidiomata omphalinoid, collybioid or gymnopoid. Pileus 10–40 mm broad, membranous, hemispheric when young, becoming convex, plano-convex to applanate, generally umbilicate to sometimes slightly depressed at the centre, inflexed then straight or reflexed at margin, with a marginal zone often undulating with age, glabrous, radially striate or grooved towards the margin, orange (6B7) or reddish orange (7B7) to brown (7D8) overall when young, somewhat reddish orange (7B7) or dark brown (7F8), then paler towards the margin, white or pale orange (6A3) to light brown (6D4), often greyish orange (6B4) to dark brown (6F8) at the disc. Lamellae adnate, broad, ventricose to broadly ventricose, white when fresh, sometimes with greyish red (7B4) to brown (7E7) tint somewhere, margin entire to split and sometimes grooved, L = 12–17, l = 3–5. Stipe 10–30 mm long, 2–4 mm thick in the middle, central, cylindrical, or compressed, with dense basal mycelium when young that disappears when old, hollow, fibrous, glabrous, slightly longitudinally striate when old, rooting deep in the substrate, but eventually attaches to the stump, dull white to greyish red (7B4) when young, soon darker towards the base, white to reddish orange (7A7) at apex, finally entirely dark brown (7F8). Odour not distinctive.

**Figure 2.**
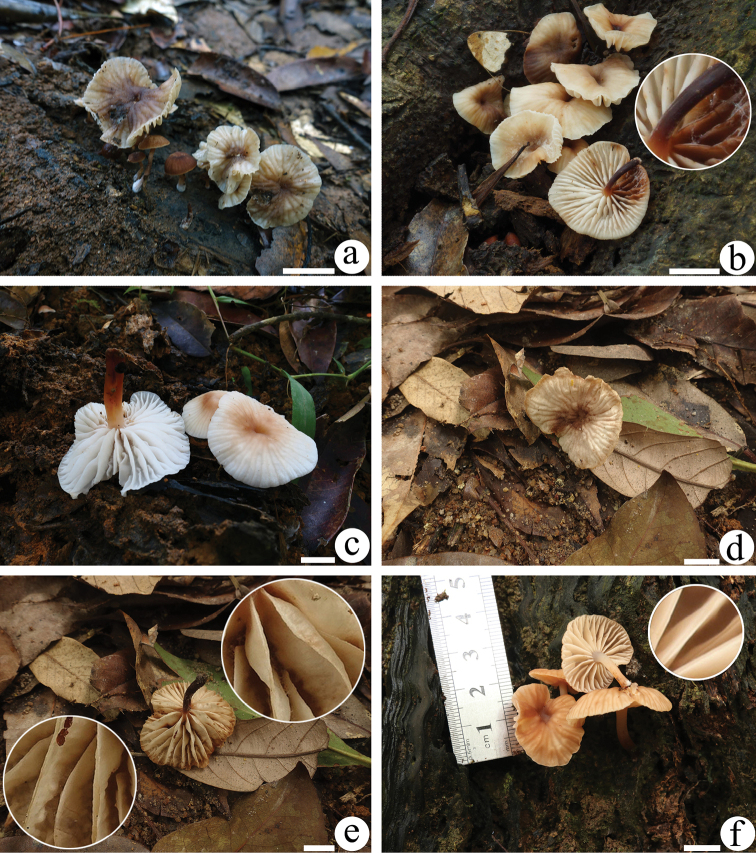
Basidiomata of *Gymnopusomphalinoides***a**GDGM 78483 **b**GDGM 78318 holotype! (with magnifying slightly longitudinally striate stipe) **c**KUN-HKAS 107312 **d, e**GDGM 44411 **f**HMJU 00506. **a** photographed by M. Zhang **b** photographed by L.Q. Wu, **c** photographed by X.H. Wang **d, e** photographed by J.P. Li **f** photographed by J.Z. Xu. For a detailed display, the slightly longitudinally striate stipe is magnified in **b**, and the split lamellar edge is magnified in **e, f**. Scale bars: 1 cm.

**Figure 3.**
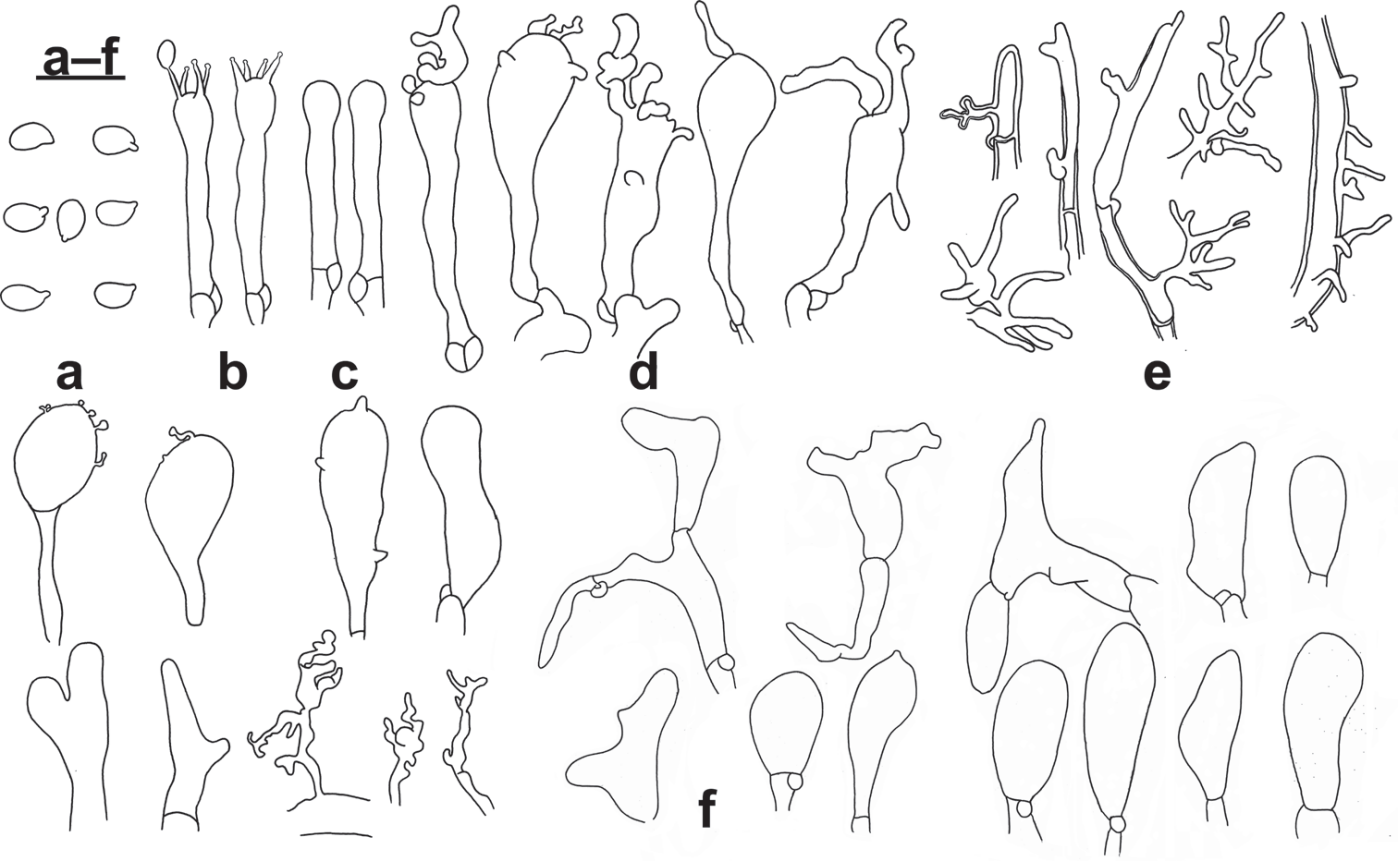
Microscopic features of *Gymnopusomphalinoides* (GDGM 78318, holotype!) **a** Basidiospores **b** Basidia **c** Basidioles **d** Cheilocystidia **e** Stipitipellis **f** terminal elements of the pileipellis. Drawing by J.P. Li. Sale bars: 10 μm (**a–d**), 20 μm (**e, f**).

Basidiospores [n=80] (3.5–) 4.0–5.5 (–6.0) × 2.5–3 (–3.5) μm (average= 4.63 × 2.93 μm, E = 1.33–1.83 (–2), Q=1.58), obovoid, ellipsoid to subellipsoid, sometimes amygdaliform. Basidia [n=20] 17–31 × 3–5 μm, clavate, 4-spored. Basidioles [n=20] 17–32 × 4–5.5 μm, clavate, cylindrical. Lamellar edge sterile. Cheilocystidia [n=20] 17–32 × 4–10 μm, irregularly clavate, sphaeropedunculate or almost so, with tendency to be inflated, with or without finger-like apical projection(s) or more or less diverticulate elements. Pileipellis a cutis composed of cylindrical, thin-walled hyphae, up to 12.5 μm wide, smooth or with scattered diverticula, hyaline to slightly brownish; *Rameales*-like structures present, rare to abundant; terminal cells short, broad, mostly inflated, vesiculose or pyriform to cystidioid (clavate), obtuse and sometimes diverticulate, mixed with a few irregularly branched, slightly coralloid elements and some resembling *Dryophila*-type structures. *Stipitipellis* a cutis composed of cylindrical, slightly thick to thick-walled, smooth, non-dextrinoid, parallelly arranged hyphae, up to 12 μm wide, with or without *Rameales*-like structure. Caulocystidia absent. Clamp connections present.

#### Ecology.

Saprotrophic, gregarious or in small clusters, usually rooting around the roots and stumps in broadleaf forests.

#### Additional specimens examined.

China, Guangdong Province, Guangzhou City, Tianluhu Forest Park, longitude and latitude not recorded, alt. not recorded, 4 April 2019, T.H. Li, W.Q. Deng, J.Y. Xu & J.P. Li (GDGM 44411); Guizhou Province, Tongren City, Fanjingshan National Nature Reserve, 27°48'33"N, 108°44'45"E, alt. 640 m, 14 July 2019, J.Z. Xu (HMJU 00506); Yunnan Province, Pu’er City, Meizihu Reservoir, 22°45'0"N, 100°58'48"E, alt. 1300 m, 19 September 2019, M. Zhang, T. Li & J.Y. Xu (GDGM 78483); Yunnan Province, Maguan County, Nanlao Village, 23°03'21"N, 104°31'12"E, alt. 1190 m, 5 August 2017, X.H. Wang (KUN-HKAS 107312).

#### Remarks.

*Gymnopusomphalinoides* is a very distinct species due to its generally omphalinoid basidiomata, by a membranous and striate or grooved, reddish brown to brown pileus that becomes paler with age, by the broad, adnate, ventricose lamellae that are sometimes split to grooved at the edge, and by a pileipellis often with scattered cystidioid (clavate) or vesiculose to pyriform terminal elements. Collection GDGM 78318 is characterised by having cheilocystidia with more or less finger-like apical projection(s) and by a pileipellis with scattered *Rameales*-like structures, but the collection GDGM 44411 differs in its cheilocystidia with diverticulate elements and pileipellis with more *Rameales*-like structures.

Among the known species of *Gymnopus* with a striate or grooved pileus and ventricose lamellae, *G.bisporus* (J. Carbó & Pérez-De-Greg.) J. Carbó & Pérez-De-Greg., *G.dentatus* Murrill, *G.discipes* (Clem.) Murrill, *G.dysosmus* Polemis & Noordel., *G.fuscotramus* Mešić, Tkalčec & Chun Y. Deng, *G.pubipes* Antonín, A. Ortega & Esteve-Rav. and *G.volkertii* are similar to the new species. However, *G.bisporus*, belonging to sect. Levipedes, has a brown to reddish brown pileus and larger basidiospores (9.0–11 × 4.5–5.5 μm), and true cheilocystidia are absent (Antonín and Noordeloos 2010); *G.dentatus* has a dentate pileus margin, a white stipe and larger basidiospores (7–8.5 × 6–7 μm), growing on lawns (Murrill 1916); *G.discipes* has free lamellae and a white stipe arising from a hypogaeous disk (Murrill 1916); *G.dysosmus*, sect. Impudicae, has garlic-smelling basidiomata, dark greyish brown lamellae, larger basidiospores (8.0–11 × 3.3–4.5 μm), and caulocystidia (Antonín and Noordeloos 2010); *G.fuscotramus*, belonging to sect. Vestipedes [= *Marasmiellusfuscotramus* (Mešić, Tkalčec & Chun Y. Deng) J.S. Oliveira], has abundant rhizomorphs, larger basidiospores (8.2–9.6 × 3.7–4.4), and pale grey-brown lamellar and pileus trama (Mešić et al. 2011); *G.pubipes*, sect. Levipedes, has deeply emarginate to adnexed lamellae and an entirely pubescent stipe with numerous caulocystidia (Antonín and Noordeloos 2010); and *G.volkertii* has a umbonate and estriate pileus, adnexed lamellae, and larger basidiospores (8.2–9.6 × 3.7–4.4 μm), growing on lawn (Murrill 1916).

### ﻿ Gymnopus schizophyllus

Taxon classificationFungiAgaricalesOmphalotaceae

J.P. Li, T.H. Li & Y. Li, sp. nov.

5ABFA379-DEFA-56DB-9EA4-A453DA5DB960

837642

Figs 4 , 5

#### Typification.

China, Guangdong Province, Xinyi City, Yunkaishan National Nature Reserve, 22°17'08"N, 111°12'47"E, alt. 1453 m, 26 July 2019, B. Song, H.S. Wen & J.P. Li (GDGM 77165, holotype!).

#### Etymology.

The epithet “*schizophyllus*” (Lat.) refers to the split edge of lamellae which is not so common in the genus.

#### Diagnosis.

Differs from *G.omphalinoides* in its more or less depressed to slightly umbilicate pileus and more often split lamellar edge. Basidiomata mainly gregarious on decayed wood in broadleaf forest; pileus often pale orange to light brown; lamellae, adnate and generally split at the edge; stipe glabrous.

#### Description.

Basidiomata gymnopoid or collybioid. Pileus 10–20 mm broad, membranous, hemispherical when young, then convex, with slightly inflexed margin, expanding to plano -convex , with a depressed disc, undulating at the margin, glabrous, radially striate or grooved towards the margin, often pale orange (6A3) to light brown (6D8), darker at the centre, sometimes to dark brown (6F8), white to light brown (6D8) towards the margin. Lamellae adnate, linear to arcuate, sometimes furcate to branched or venose, generally split at the edge, dull white to brownish orange (7C7), pale at the edge, sometimes with brown (7E8) to dark brown (7F8) tints somewhere, L = 10–20, l = 3–4. Stipe 11–21 mm long, 0.8–1 mm thick in middle, central, cylindrical, straight or sometimes curved, insititious, hollow, fibrous, glabrous, rooting deep in the substrate, but eventually attaches to the stump, white to orange-white (6A2) at first, slightly darker at base, then darker towards the apex, finally entirely light brown (7D8) to brown (7E8). Odour not distinctive.

Basidiospores [n=80] 4–6 (–6.5) × 2.5–3 (–3.5) μm (average = 4.90 × 2.93 μm, E = (1.29–) 1.33–2.00 (–2.20), Q = 1.68) or [n=20] 6.5–8 × 2.5–3 μm (average = 7.35 × 2.86 μm, E = 2.17–3.2, Q = 2.65), obovoid, ellipsoid to subellipsoid, sometimes amygdaliform. Basidia [n=20] 15–32 × 4–6 μm, clavate, 4-spored, rarely 1–3-spored. Basidioles [n=20] 17–27.5 × 4–6.5 μm, clavate, cylindrical. Lamellar edge sterile. Cheilocystidia [n=20] 20–43 × 4.5–9 μm, irregularly clavate, tending to inflated, with finger-like apical projection(s) or more or less diverticulate elements. Pileipellis a cutis composed of thin-walled, cylindrical hyphae up to 18 μm wide, smooth or with scattered diverticula, hyaline to slightly greyish; *Rameales*-like structures present but very few; terminal elements short, broad, mostly inflated, vesiculose or pyriform to cystidioid (clavate), obtuse and sometimes diverticulate, mixed with a few irregularly branched elements, some resembling *Dryophila*-type structures. Stipitipellis a cutis composed of cylindrical hyphae, up to 19 μm wide, thin- to thick-walled, smooth, non-dextrinoid, diverticulate, parallelly arranged. Caulocystidia absent. Clamp connections present.

#### Ecology.

Saprotrophic, gregarious or in small clusters, usually rooting around roots and stumps in broadleaf forests.

#### Additional specimens examined.

China, Guangdong Province, Xinyi City, Yunkaishan National Nature Reserve, 22°17'10"N, 111°12'50"E, alt. 1450 m, 26 July 2019, B. Song, H.S. Wen & J.P. Li (GDGM 77038); Guangdong Province, Xinyi City, Yunkaishan National Nature Reserve, 22°17'06"N, 111°12'51"E, alt. 1450 m, 29 May 2019, B. Song, H.S. Wen & J.P. Li (GDGM 76287); Yunnan Province, Maguan County, Laojunshan Moutain, 22°56'49"N, 104°32'44"E, alt. 1960 m, 11 August 2016, X.H. Wang (KUN-HKAS 96494).

#### Remarks.

*Gymnopusschizophyllus* is a very distinct species by the orange to brown pileus that becomes paler with age; by the lamellae with generally split edge; by the two sizes of basidiospores: 1) 4–6 (–6.5) × 2.5–3 (–3.5) μm from the usual 4-spored basidia and 2) a few larger basidiospores up to 8 μm long from the 1–3-spored basidia; and by a pileipellis often with scattered cystidioid (clavate) or vesiculose to pyriform terminal elements.

Morphologically, among the known species of *Gymnopus* with a striate or grooved pileus and similarly sized basidiospores, *G.discipes*, *G.expallens* (Peck) Murrill, *G.fusipes* (Bull.) Gray, *G.micromphaloides* R.H. Petersen & K.W. Hughes, *G.oculatus* Murrill, *G.omphalinoides*, *G.pseudomphalodes* (Dennis) J.L. Mata, *G.purpureicollus* (Corner) A.W. Wilson, Desjardin & E. Horak, *G.sepiiconicus* (Corner) A.W. Wilson, Desjardin & E. Horak and *G.subflavescens* Murrill are similar to the new species. However, *G.discipes* has a subfleshy pileus with a wide umbo, free and ventricose lamellae and a white stipe (Murrill 1916); *G.expallens* has basidiomata with a distinct odour, a hygrophanous pileus, adnexed and ventricose lamellae, and a broad stipe up to 4 mm (Murrill 1916); *G.fusipes* has a fleshy pileus and a fusoid stipe with pseudorrhiza (Antonín and Noordeloos 2010); *G.micromphaloides*, sect. Vestipedes [= *Collybiopsismicromphaloides* (R.H. Petersen & K.W. Hughes) R.H. Petersen], has adnexed and ventricose lamellae, a scurfy-vestured stipe, and strongly encrusted hyphae of the pileipellis (Petersen and Hughes 2014); *G.oculatus* has a white pileus in general, nearly free lamellae and a whitish pruinose, larger stipe (Murrill 1916); *G.omphalinoides* generally has a deeply umbilicate pileus, broad, adnate and ventricose lamellae; *G.pseudomphalodes* has a cream pileus and regularly cylindrical cheilocystidia (Dennis 1961); *G.purpureicollus* has a hygrophanous pileus, subfree to adnate lamellae with a decurrent tooth and a lamellar edge without cheilocystidia (Wilson et al. 2004); *G.sepiiconicus*, sect. Levipedes, has hyphae with annular incrustations in the stipitipellis (Wilson et al. 2004); and *G.subflavescens* has white basidiomata overall, crowded lamellae and small, globose basidiospores (Murrill 1916).

**Figure 4.**
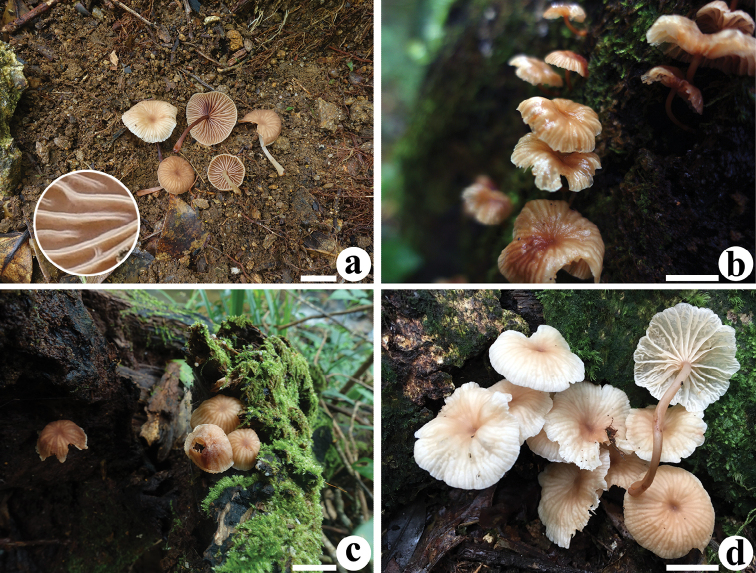
Basidiomata of *Gymnopusschizophyllus***a**GDGM 77038 **b**GDGM 76287 **c**GDGM 77165 holotype! **d**KUN-HKAS 96494 **a, c** photographed by J.P. Li **b** photographed by H.S. Wen **d** photographed by S.H. Li. For a detailed display, the split lamellar edge is magnified in a. Scale bar: 1 cm.

**Figure 5.**
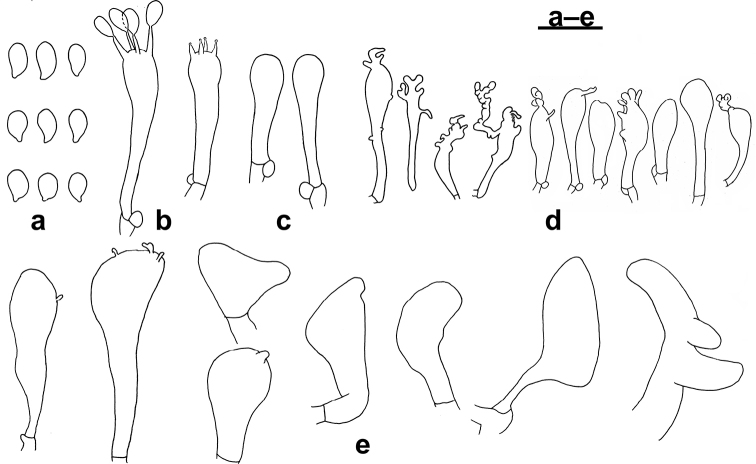
Microscopic features of *Gymnopusschizophyllus* (GDGM 77165, holotype!) **a** Basidiospores **b** Basidia **c** Basidioles **d** Cheilocystidia **e** terminal elements of the pileipellis. Drawing by J.P. Li. Scale bars: 10 μm (**a–c**), 20 μm (**d, e**).

## ﻿Discussion

According to the phylogenetic results, the two new species could be taken to represent a new section within Gymnopus s. s.tr., a new subsection of Gymnopussect.Gymnopus or a new member of G.sect.Gymnopus. Suppose the two new species and samples from India represent a new section or subsection? In that case, the samples from New Zealand may occupy a taxonomic position at the same level due to their phylogenetic relationship. Thus, given the three alternative systematic interpretations for the two new species and the monophyletic group they form, we argue that the morphological features and evidence from the molecular data strongly support the two new species as members of G.sect.Gymnopus.

**Figure 6.**
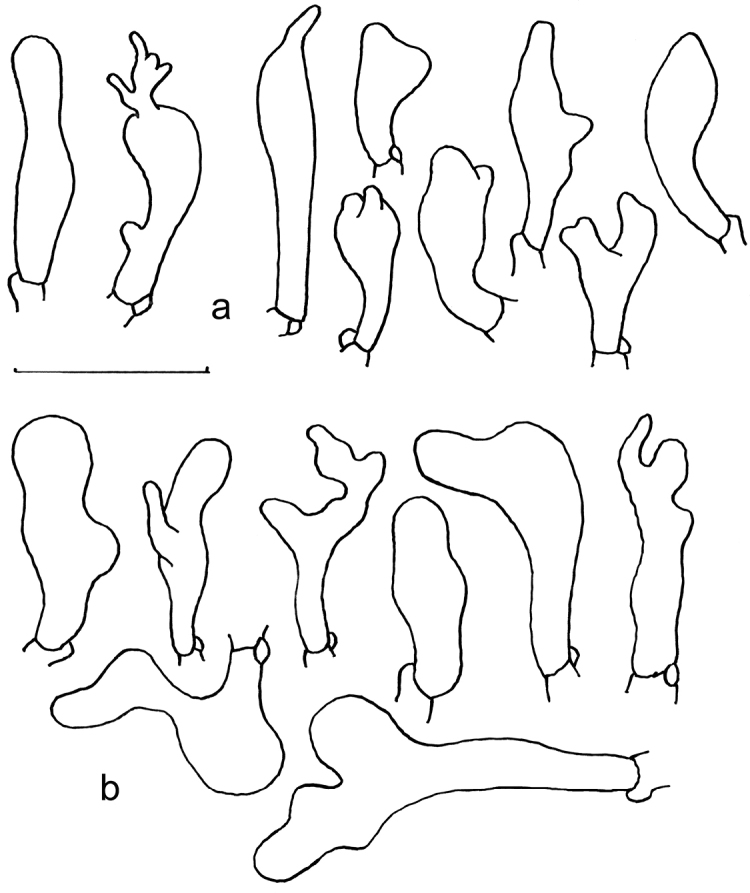
*Gymnopusfusipes* (Mokrá near Brno, place called Nad dlouhým (Sivický les forest), 18 June 2002, A. Vágner, BRNM 670783) **a** Cheilocystidia **b** Pileipellis terminal cells. Drawings by V. Antonín. Scale bar: 20 µm.

Morphologically, the taxonomic placement of *G.omphalinoides* and *G.schizophyllus* can be correlated with the pileipellis features, particularly its terminal cells. After comparison, the two new species with glabrous stipe and at least the part of *Dryophila*-like structures in pileipellis are easily confused with species within the G.sect.Levipedes (Fr.) Halling (Antonín and Noordeloos 2010). However, the new species have additional inflated and broad pileipellis terminal elements and are only distantly related to that section. Gymnopussect.Androsacei and G.sect.Gymnopus are included in a strongly supported clade, indicating they are close. But G.sect.Androsacei has rhizomorphs, dextrinoid trama (at least in the stipe apex) and a pileipellis mixed with broom cells (Antonín and Noordeloos 2010). Furthermore, G.sect.Androsacei does not form a distinct monophyletic clade neither in this study nor in Oliveira et al. (2019), César et al. (2020), and so forth. This issue needs to be addressed in future studies. Currently, known species with molecular data are very few, which perhaps could explain this topologic structure. Additionally, a phylogenetic tree based on more genetic markers might provide an improved result. Besides, G.sect.Impudicae is characterised by basidiomata with distinctive odour and often inconspicuous cheilocystidia (Antonín and Noordeloos 2010). These divergent morphological features reflect the non-trivial phylogenetic distance from the two new species. Unexpectedly, the two new species have a membranous pileus and non-fusoid stipe devoid of pseudorrhiza, contrary to the traditional circumscription of G.sect.Gymnopus in macro-morphology. However, the molecular phylogenetic results reveal that the clade they form is the most closely related group to G.sect.Gymnopus except for the two samples from New Zealand. After examining the micromorphological structures intensively, the synapomorphy eventually came to the surface. Cheilocystidia of both newly described species are versiform diverticulated cells and generally agree in size and shape with those of *G.fusipes* (Fig. 6). Also, the pileipellis, composed of inflated elements with some resembling *Dryophila*-type structures, is similar to *G.fusipes* and follows the key rule for sectional delimitation in *Gymnopus* s. str. [for a detailed macro- and micromorphological description of *G.fusipes* see Antonín and Noordeloos (1997, 2010)]. Besides, the two new species lack a typical *Rameales*-type pileipellis and any well-developed caulocystidia, in contrast to G.sect.Vestipedes which is already a part of *Collybiopsis* (Antonín and Noordeloos 2010; Oliveira et al. 2019; Petersen and Hughes 2021). Furthermore, the original G.sect.Perforantia is currently considered a distinct genus – *Paragymnopus* – whose members usually have non-glabrous stipe and lack cheilocystidia (Petersen and Hughes 2016; Oliveira et al. 2019).

As the characteristic of the pileipellis is a significant factor for sectional delimitation in *Gymnopus*, the features in macro-morphology are second. The current sectional concept was summarised based on features from one species, *G.fusipes*. That means the single known species circumscribes the current knowledge at the sectional level. This is also why only minor divergence in micro-morphology occurs between G.sect.Gymnopus and the two new species. Following the indication from phylogenetic results and similarity of micro-morphology, thus, an emended and improved concept of G.sect.Gymnopus is proposed herein by including *G.omphalinoides* and *G.schizophyllus*.

A very interesting and unusual characteristic is a splitting lamellar edge in both newly described species. What advantage such split lamellar edge could confer is difficult to surmise, but Antonín and Herink (1999) described the same characteristic in *Gymnopusluxurians* (Peck) Murrill [recently *Collybiopsisluxurians* (Peck) R.H. Petersen]. They proposed that this may be a reaction to specific climatic conditions (the higher humidity, the better hymenium development) because it was most distinct in the collections from greenhouses, botanic gardens and tropical Africa.

Borthakur and Joshi (2016) provided a nrITS sequence and a few morphological characteristics of the collection NEHU MBSRJ48 annotated as *Micromphalefoetidum* which comes from a subtropical forest of Northeast India, quite similar to *G.schizophyllus.* However, the sequence is quite different from the sequences more well-recognised for the current *Gymnopusfoetidus* (Sowerby) P.M. Kirk. It likely represents an incorrectly determined ITS sequence in GenBank like several others as argued by Nilsson et al. (2006) and Hofstetter et al. (2019). The specimen has a depressed to umbilicate pileus, a glabrous stipe and similarly sized basidiospores (5.2 × 2.88 μm). The nrITS sequence is highly similar to that of *G.schizophyllus*, implying they are possibly conspecific. The collection from India clearly belongs in G.sect.Gymnopus. The collections from New Zealand, named as *Marasmiusotagensis*, are characterised by a depressed to umbilicate pileus, glabrous stipe and a pileipellis with broad, mostly inflated terminal elements (according to photos from Dr. Jerry Cooper). The phylogenetic placement indicates that this is another member of G.sect.Gymnopus.

### ﻿ Gymnopus sect. Gymnopus

Taxon classificationFungiAgaricalesOmphalotaceae

, emend.

EE9234FB-C188-5CCD-B0A3-8C7B233F0CC5

#### Emended circumscription.

Pileus membranous or fleshy; stipe smooth or slightly to deeply sulcate-striate, with a well-developed or reduced pseudorrhiza; spore print white to pale ochraceous; cheilocystidia versiform, clavate, fusoid, tending inflated, sometimes with more or less finger-like apical projection(s), or diverticulate elements; pileipellis a cutis, or this transitioning to a trichoderm, with broad terminal elements, mostly inflated, mixed with irregularly branched elements and some resembling *Dryophila*-type structures; no dextrinoid or cyanophilous structures; rooting in the substrate, frequently on roots or stumps.

**Type species.***Gymnopusfusipes* (Bull.) Gray

**Other currently recognised species.***G.omphalinoides* J.P. Li, T.H. Li & Y. Li, *G.schizophyllus* J.P. Li, T.H. Li & Y. Li

### ﻿A key to species of Gymnopussect.Gymnopus

**Table:** 

1	Pileus fleshy; stipe with a distinct pseudorrhiza	***G.fusipes***
–	Pileus membranous; stipe without a pseudorrhiza but rooting in the substrate	**2**
2	Pileus generally deeply umbilicate; lamellae broad, adnate and ventricose	***G.omphalinoides***
–	Pileus more or less depressed; lamellae adnate, linear to arcuate	***G.schizophyllus***

## Supplementary Material

XML Treatment for Gymnopus omphalinoides

XML Treatment for Gymnopus schizophyllus

XML Treatment for Gymnopus sect. Gymnopus
